# Protective Effects of *α-*Tocopherol on ABR Threshold Shift in Rabbits Exposed to Noise and Carbon Monoxide

**Published:** 2011

**Authors:** Masoud Motallebi Kashani, Seyyed Bagher Mortazavi, Ali Khavanin, Abdolamir Allameh, Ramezan Mirzaee, Mehdi Akbari

**Affiliations:** a *Department of Occupational and Environmental Health, School of Medicine, Tarbiat Modrres University, Tehran, Iran*; b *.Department of Biochemistry, School of Medicine, Tarbiat Modarres University, Tehran, Iran. *; c *Department of Occupational Health, School of Health, Zahedan University of Medical Sciences, Zahedan, Iran. *; d *Department of Audiology, School of Rehabilitation Sciences, Iran University of Medical Sciences, Tehran, Iran.*

**Keywords:** Noise induced hearin gloss, Anti oxidant agent, NIHL potentiation, Auditory brainstem response, *α*-Tocopherol

## Abstract

Noise induced hearin gloss (NIHL) is one of the most important occupational disease world wide. NIHL has been found potentiate by simultaneous carbon monoxide (CO) exposure. Free radicals have been implicated in cochlear damage resulted from the exposure to noise and due to the CO hypoxia. This study examined whether *α*-tocopherol administration , as a free radical scavenger, causes the attenuation of auditory brainstem response (ABR) threshold shifts resulting from noise exposure and noise plus CO exposure. Forty-two rabbits were divided in to seven groups including control, noise + saline, noise + CO + saline, noise + *α*-tocopherol, noise + CO + *α*-tocopherol , CO + *α*-tocopherol and *α*-tocopherol alone. ABR was assessed before exposure, 1 hand 14 days post exposure. The administration of 50 mg/Kg of *α*-tocopherol prior, following and post exposure to noise or noise plus CO recovered permanent ABR threshold shift at 1 and KHz almost to the baseline and provided significant attenuation in permanent ABR threshold shift at 4 and 8 KHz in subject swhich were exposed to noise but it did not block the potentiating of threshold elevation by CO exposure (extra threshold loss by combined exposure) at 4 and 8 KHz. *α* Tocopherol provides protective effect against the hearing loss resulting from noise exposure and simultaneous exposure to noise plus CO.

## Introduction

Noise induced hearing loss (NIHL) is a widespread disease in the world with 16% of the disabling hearing loss in adults (over 4 million DALYs) being attributed to the occupational noise (1). Simultaneous exposure to noise and carbon monoxide (CO) result in greater NIHL, beyond which is produced by noise alone, while CO exposure alone may not induce permanent hearing loss (2). CO is the most abundant pollutant in air and is present along with noise in many work environments. In many cases, a reduction of noise intensity and CO concentration in the workplace is very difficult; however, personal protective equipment is seldom used by workers because of inherent limitations. Hence, a pharmacological preventative measure for NIHL can be an important element of hearing conservation programs in the workplace.

Previous researches have shown the essential role of free radical formation in the cochlea in NIHL (3, 4). Early reports of noise-induced free radical formation in the cochlea led to the hypothesis that NIHL can be attenuated by exogenous antioxidants as well as endogenous reactive oxygen species (ROS) scavengers. Many compounds have been used for the prevention and treatment of NIHL (5, 6). The mechanism underlying NIHL potentiation by CO is unclear but combined exposure to noise and CO is believed to increase free oxygen radical levels in the cochlea (7, 8). 

It is indicated that *α*-tocopherol, as an antioxidant with no noticeable side effects, can effectively attenuate the noise-induced ABR Threshold shift in guinea pigs (9).

This study has been designed to test the hypothesis that injury resulting from the noise exposure and simultaneous exposure to noise plus CO is reduced by *α*-tocopherol.

## Experimental

*Animals*

Healthy male adult white New Zealand rabbits (2200-2500 g body weight) were acquired from the Pasture Institute of Iran. Animals were examined for possible otitis media prior to auditory assessments. Rabbits were maintained on a light-dark cycle (12 h-12 h) at 23-27^°^C and had free access to food (Purina laboratory chow) and water. All procedures regarding the use and handling the animals were carried out in accordance with the principles of Helsinki and in accordance with animal use protocols approved by the appropriate institutional animal care and use committees.

*Experimental protocol*

Forty-two rabbits were divided into seven groups (6 per group (n = 6)) according to the type of experiment as follows: Group 1: Control (no exposure to noise or CO, and no IP injection), Group 2: Exposed to the noise alone and received saline IP injection, Group 3: Exposed to the noise plus CO and received saline IP injection, Group 4: Exposed to the noise alone and received *α*-tocopherol 50 mg/Kg body weight IP injection, Group 5: Exposed to the noise plusCO and received *α*-tocopherol 50 mg/Kg body weight IP injection, Group 6: Not exposed to the noise or CO and received α-tocopherol 50 mg/Kg body weight IP injection, Group 7: Exposed to CO alone and received saline IP injection.

The total duration of experimental procedures was 22 days. Noise and CO exposure was conducted for 5 consecutive days on 4^th^ day through 8^th^ day. α-Tocopherol or saline was injected once a day from the 1^st^ day through the 11^th^ day (3 days before the exposure, 5 days during the exposure and 3 days after the exposure). Auditory brain-stem response (ABR) threshold at 1, 2, 4 and 8 KHz were measured at three time points: 1^st^ day (prior to experiment), 8^th^ day (immediately at the end of the exposure) and 22^nd^ day (14 days after the exposure).

The exposure chamber was designed as a reverberant field which could accommodate six rabbits simultaneously. The chamber was equipped with stereo speakers for delivering the noise. Air exchange rate in this chamber was adjusted to 12 air changes per hour using an exhaust fan. The ventilation system generated noise below 40 dB which was masked by the noise generated for exposure experiments. Experimental groups 2, 3, 4 and 5 were exposed to octave band noise centered at 4 KHz, 100 dB SPL, 8 h per day for 5 days consecutively. This octave band noise was generated and controlled with a custom software program (signal software) and then amplified and delivered with a computer system using Cool Edit software to the two load speakers. Sound pressure level in the chamber was measured using a precision sound level meter (Cel-460, UK) at the level of animal head (15 cm above the chamber floor). Noise exposure levels in the chamber were monitored every 30 min. The variability of the noise within the animals’ roaming area was less than 2 dB.

Animals exposed to the noise plus CO or CO alone based on the experimental design. The CO concentration in the exposure chamber was 700 ppm with an actual level achieved by 700 ± 25 ppm (SD). Previous studies have shown that carboxyhemoglobin levels approach steady state within 30 min of exposure onset and have stabilized after 90 min of exposure (2). Therefore, CO exposure began 90 min prior to the onset of noise to assure the equilibration of carboxyhemoglobin. The total daily exposure time for noise was 8 h and the daily exposure time for CO was 9.5 h (8 + 1.5 h). Experimental groups 3, 5 and 7 were exposed to CO at 700 ppm concentration, 9.5 h per day for 5 days consecutively. CO gas was delivered into the chamber using a micro valve (Air Flow) and the CO level in the chamber was measured according to a standard procedure described in NIOSH (Method No. 6604 by carbon monoxide monitor STICK (testo 317-3)) (10). The concentration of CO was monitored continuously. Experimental groups 4, 5 and 6 received *α*-tocopherol ( ( + ) - *α*-tocopherol, mixed isomers, 1013 IU vitamin E/g, Sigma) 50 mg/Kg body weight once a day for 11 days by IP injection. During the exposure days and *α*-tocopherol was given 1 h prior to noise or noise plus CO exposure. Groups 1, 2, 3 and 7 received saline with a similar volume and schedule. Protective effect of *α*-tocopherol against CO alone was not studied since CO alone does not cause any permanent auditory impairment (2).

*Auditory brainstem response testing*

Auditory function was evaluated by auditory brainstem response (ABR) threshold test. Before ABR test, animals were anesthetized with Xylazine (10 mg/Kg body weight) and ketamine (40 mg/Kg body weight) mixture by intramuscular (IM) injection. The external ear canals and tympanic membranes were inspected to ensure the ear canal to be free of wax. The body temperature of rabbits was maintained at 37 ± 1^°^C using a heating blanket. An active needle electrode was inserted subcutaneously below the test ear, a reference electrode at the vertex and a ground electrode below the other ear. The test stimuli were 1, 2, 4 and 8 KHz tone bursts (1 msec Blackman rise/fall, 15 msec duration, alternating polarity) generated using Interacoustics EP25. Stimuli were routed through an insert phone fitted in the rabbit’s ear canal. Near the threshold, the sound intensity was decreased in 5-dB steps and responses for 1024 tone presentations at 20/s were averaged at each intensity level. ABR threshold was defined as the lowest stimulus intensity that produces a reproducible ABR waveform peak 3 or 4. The average threshold of the right and left ear at a specific frequency was considered as the ABR threshold for a specific time point. All experimental groups were tested prior to the experiment on the 1^st^ day to establish the baseline ABR threshold, on the 11^th^ (immediately at the end of exposure) and 22^nd^ day (14 days after the exposure). Temporary threshold shift (TTS) was calculated at each frequency as the difference between baseline ABR threshold and ABR threshold on 11^th^ day (0 day post exposure). The difference between baseline ABR threshold and ABR threshold at each frequency on the 22^nd^ day was considered as the permanent threshold shift (PTS).

*Biochemical assay*

Biochemical measurement was performed to evaluate the effects of *α*-tocopherol on the rabbits’ blood antioxidant activity. Ferric reducing ability of plasma (FRAP assay) was used to compare the antioxidant capacity of the plasma from control and treated rabbits. Blood samples (2 mL) were collected from the heart into heparin zed tubes and centrifuged at 1500 g for 10 min. Plasma antioxidant activity was measured using the FRAP assay as described previously by Benzi and Strain (11).This assay was performed at two time points: 4^th^ day (3 days after receiving *α*-tocopherol or saline and prior to noise or CO exposure), and 8^th^ day (8 days after receiving *α*-tocopherol or saline and 5 days post exposure to noise or noise plus CO).

*Statistical analysis*

The Kolmogorov-Smirnov test (K-S test) was used to assess the data normality in each group. The ABR threshold shift at each frequency was compared among groups using one-way analysis of variance (ANOVA) and the Tukey test was applied as a post-hoc test for multiple comparisons among the groups. Likewise, ANOVA was used for the comparison of ABR threshold shift among the frequencies at each group and the comparison of FRAP level among the groups. All statistical tests were performed using SPSS 15 and p < 0.05 was considered significant.

## Results

*FRAP assay*

**Figure 1:**
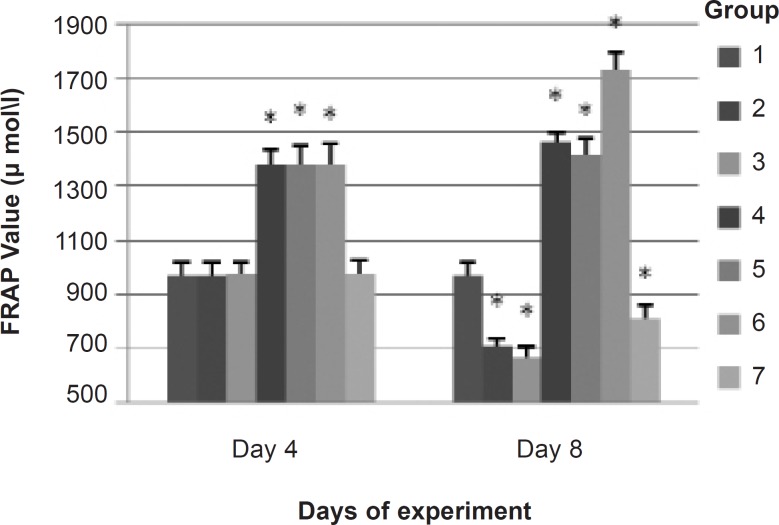
Total antioxidant ability of plasma on 4^th^ (prior to exposure) and 8^th^ day (5 days after exposure) in each group Bars show means, error bars show ± 1.0 SD. * p < 0.05, significantly different from group 1. 1: control (no exposure to noise or CO, and no IP injection); 2: noise exposure + saline IP injection; 3: noise plus CO exposure + saline IP injection; 4: noise exposure + *α*-tocopherol 50 mg/Kg IP injection; 5: noise plus CO exposure + *α*-tocopherol 50 mg/Kg IP injection; 6: no exposure to noise or CO + *α*-tocopherol 50 mg/Kg IP injection; 7: CO exposure + saline IP injection

The results from the FRAP assay indicated that the total antioxidant capacity of plasma from the groups which received *α*-tocopherol (groups 4, 5 and 6) was significantly more than the other groups on 4^th^ day (prior to exposure) and 8^th^ day (5 days after the exposure). FRAP level on 8^th^ day was found to be reduced significantly in groups 2, 3 and 7 as compared to group 1 (control). Likewise, FRAP level in group 3 was significantly less than group 2 (Figure 1).

*ABR measurement*

**Figure 2:**
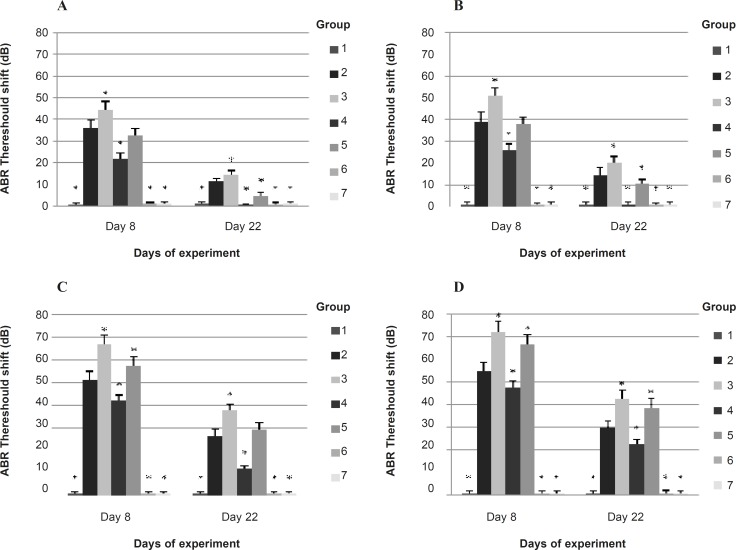
ABR threshold shifts on 8^th^ day (1 h post exposure to noise or noise plus CO ) and 22^nd^ day (14 days post exposure to noise or noise plus CO ) in each group at (A) 1 KHz, (B) 2 KHz, (C) 4 KHz and (D) 8 KHz Bars show means, error bars show ± 1.0 SD. * p < 0.05, significantly different from group 2. 1: control (no exposure to noise or CO, and no IP injection); 2: noise exposure + saline IP injection; 3: noise plus CO exposure + saline IP injection; 4: noise exposure + *α*-tocopherol 50 mg/Kg IP injection; 5: noise plus CO exposure + *α*-tocopherol 50 mg/Kg IP injection; 6: no exposure to noise or CO + *α*-tocopherol 50 mg/Kg IP injection; 7: CO exposure + saline IP injection

Baseline ABR thresholds at each frequency were similar in all groups. The baseline ABR threshold at 8 and 4 KHz were significantly smaller than 2 and 1 KHz in all groups. ABR threshold shift on 8^th^ day (1 h after the exposure) and 22^nd^ day (14 days after the exposure) at each frequency in experimental groups are represented in Figure 2.

The ABR threshold shift of the control group was approximately 0 on 8^th^ and 22^nd^ days. Statistical comparisons showed that the ABR threshold shifts of the 6^th^ group (unexposed and received *α*-tocopherol) and the 7^th^ group (CO exposure and received saline) at each frequency, were not significantly different from that of the 1^st^ group (control) at all time points. This indicates that CO exposure alone and receiving *α*-tocopherol alone could not alter ABR thresholds. The ABR threshold of the 2^nd^ group (noise + saline) had significant elevation compared to the control group at all frequencies on 8^th^ and 22^nd^ days but was significantly smaller than the ABR threshold of the 3^rd^ group (noise + CO + saline) (p < 0.01).

The saline + noise group (group 2) had a 44.9 dB threshold loss on 8^th^ day and 20.3 dB threshold loss on 22^nd^ day averaged across frequencies of 1-8 KHz, while animals receiving saline + noise + CO (group 3) had a 58.4 dB and 27.8 dB average threshold loss on 8^th^ and 22^nd^ days, respectively. The ABR threshold shifts of 3^rd^ group at 4 and 8 KHz were significantly more prominent than at 1 and 2 KHz at all time-points tested (p < 0.01). The noise-induced ABR threshold shifts at all frequencies were found to be reduced significantly in animals which were exposed to noise and received *α*-tocopherol (group 4) compared to subjects which were exposed to noise and received saline (group 2) on 8^th^ and 22^nd^ days (p < 0.01). This reduction at 4 and 8 KHz was significantly more at 1 and 2 KHz (p < 0.01). The average threshold elevation across frequencies 1-8 KHz in the 4^th^ group was 34.1 dB on 8^th^ day and 8.6 dB on 22^nd^ day.

The ABR thresholds of animals in the 4^th^ group at all frequencies, were significantly greater than that of control group 1 h after exposure while 14 days after exposure the thresholds at 1 and 2 KHz recovered almost to the baseline.

The ABR threshold of animals exposed to noise plus CO and treated with α-tocopherol (group 5) at all frequencies was significantly smaller than that of animals which were exposed to noise plus CO and received saline (group 3) on 8^th^ day but no significant differences were found in ABR threshold shift at 8 KHz on 22^nd^ day between the 3^rd^ and the 5^th^ groups. This indicates that the treatment with *α*-tocopherol could reduce the temporary ABR threshold shift at all frequencies in subjects exposed to noise and CO simultaneously but could not protect the subjects against permanent ABR threshold shift at 8 KHz.

The ABR threshold loss of the 5^th^ group was 48.6 dB on 8^th^ day and 20.6 dB on 22^nd^ day averaged across frequencies 1-8 KHz. These values were significantly different from the control group (p < 0.01). Comparison among the experimental groups in Figure 2 suggests that while *α*-tocopherol could attenuate ABR threshold shifts in animals exposed to noise plus CO, it did not block the potentiating of threshold elevation by CO exposure (extra threshold loss by combined exposure) at 4 and 8 KHz.

## Discussion

The results showed that when *α*-tocopherol was administered 3 days prior, following post exposure to noise (or noise plus CO) by IP injection (50 mg/Kg body weight), adequate protection was provided against noise induced hearing loss and partial protection was provided against combined exposure-induced hearing loss in rabbits.

Rabbits were used as the animal model in this investigation because their hearing frequency range is approximately 360-4200 Hz, which covers the frequency range of humans. Octave band noise centered at 4 KHz, 100 dB SPL, 8 h per day for 5 days consecutively was chosen to imitate continuous noise in the work place. Likewise, maximum potentiation of NIHL by CO was reported at 100 dB SPL (12).

However, significant potentiation of NIHL by CO was reported with 500 ppm and higher concentrations of CO in a previous study (12). Preliminary experiments demonstrate that at least 700 ppm of CO exposure is required to provide more ABR threshold elevation than noise alone. Rabbits exposed to simultaneous noise and CO had further elevation in temporary and permanent threshold shift at all frequencies as compared with those exposed to noise. This phenomenon was reported in several previous studies (2, 7, 13, 14).

 Administration of *α*-tocopherol was begun 3 days before the noise (or noise plus CO) exposure for providing a stable and higher concentration of *α*-tocopherol in the plasma and in the cochlea at beginning of the exposure. FRAP assay showed that the total antioxidant capacity of plasma at groups that received *α*-tocopherol were increased as compared with other groups prior to exposure. Although there is no strong evidence to show a correlation between the oxidative damage in cochlea and the changes in the balance of oxidative stress and the antioxidant system in blood, the FRAP assay was measured as a single parameter which represents the effect of *α*-tocopherol on total antioxidant capacity of plasma and shows the changes in antioxidant level in blood during the exposure. Measurement of free radicals in the cochlea is extremely difficult because of the small size and inaccessible nature of the cochlea. In a previous study, the FRAP assay was used to measure the total antioxidant capacity of plasma in animals in response to the noise (15). However, it was indicated that α-tocopherol can somehow enter the cochlea and be used by the hair cell (16). Depletion of antioxidant capacity of plasma in animals exposed to noise may indicate that noise-induced damage is a generalized oxidative stress rather than localized one. This phenomenon was reported in previous study (15). *α*-Tocopherol was given 3 days post exposure since there is evidence for delayed free radical formation, peaking 7-10 days following noise exposure; evidence also exists on the finding that free radical scavengers administered as long as 3 days post-noise attenuate free radical formation and that they can reduce NIHL (4).

Results of this study indicate that at 1 and 2 KHz, *α*-tocopherol attenuated the temporary ABR threshold shift more significantly than at 4 and 8 KHz in subjects exposed to noise (or noise plus CO) while noise (or noise + CO ) could cause more temporary ABR threshold shift at 4 and 8 KHz than at 1 and 2 KHz. This finding is consistent with previous research showing that *α*-tocopherol, as an antioxidant, was more effective at frequencies away from the frequencies with greater threshold shift (9).This phenomenon has been reported earlier. For example, Ohainata *et al*., reported that 4 KHz octave band noise exposure produced greater threshold shifts at 4, 8, and 12 KHz than at 2, 4, and 16 KHz in guinea pigs, but glutathione supplementation attenuated the extent of threshold shifts more prominently at 2, 4 and 16 KHz (17). Yamasoba* et al*., reported that guinea pigs exposed to 4 KHz octave band noise, had maximum threshold shifts at 4 and 8 KHz, but the threshold shifts at 2, 16, and 20 KHz were significantly attenuated by supplementing deferoxamine mesylate and mannitol (18). These results may be due to the other factors, in addition to ROS formation.

The current work showed that *α*-tocopherol recovered permanent ABR threshold shift at 1 and 2 KHz almost to the baseline and provided significant attenuation in permanent ABR threshold shift at 4 and 8 KHz in subjects exposed to noise, but it could not attenuate permanent ABR threshold shift at 4 and 8 KHz in subjects exposed to simultaneous noise and CO. However, permanent ABR threshold shift were attenuated about 7.2 dB averaged across the frequencies of 1-8 KHz in this group by *α*-tocopherol. In other words, while potentiation of NIHL by CO was observed at high frequencies rather than low frequencies, *α*-tocopherol could not provide a protective effect against the potentiation.

We know that two major mechanisms are involved in NIHL including mechanical damage and metabolic alternations, but the mechanism of the potentiation of NIHL by CO is still unclear. The noise-CO interaction might not be related to the special influence of the CO on the auditory cell. It is well known that free radicals are generated during cerebral ischemia and even specifically during CO hypoxia (19). Free radicals demonstrated by EPR in focal cerebral ischemia included superoxide and peroxyl radicals in the intra-ischemic period, whereas hydroxyl radicals were observed postischemia (20). CO exposure causes reduction of oxygen supply and makes recovery of outer hair-cell function impossible, while subjects exposed to noise alone, are capable of recovery from a temporary threshold shift (2). During combined exposure, free radicals generated due to the noise exposure, in addition to free radicals generated during CO exposure, may together override endogenous free radical scavenging systems (21, 22). In other words, combined exposure to noise plus CO causes overriding inherent free radical scavenging system and resulting in irreversible cochlear damage that affect recovery of NIHL.

*α*-Tocopherol, as a potent chain-breaking lipid-soluble antioxidant, could protects the hair-cells from being damaged by cleaning the free radicals during noise exposure while the protection of *α*-tocopherol against the noise plus CO exposure-induced threshold elevation did not exceed that against the threshold elevation by the noise alone. This may be due to the CO exposure which produced sufficient damage to the outer hair-cell and hence, the recovery of function is not possible. This finding is consistent with previous studies but the current data showed that *α*-tocopherol provides more protective effect than the other compounds which were used against combined exposure (8, 23). It has been reported that the antioxidant agent vitamins A, C, and E act in synergy with magnesium to effectively prevent noise induced trauma more than the time, when these agents were delivered alone (24). Thus, combination of antioxidant agents may have more protective effects against simultaneous exposure to noise and CO. Hence, further studies with combination of antioxidant compounds were recommended. 

*α*-Tocopherol is a potent lipophilic free radical scavenger that has no noticeable side effects and has been considered to be effective clinically for the prevention of cardiovascular disease and cancer (25, 26). Therefore, *α*-tocopherol may be an excellent candidate for the prevention of NIHL in humans. It must be mentioned that these findings are limited due to the one-time point and one-dosage level administration of *α*-tocopherol. Further studies are needed to verify its action.

## Conclusion

*α*-Tocopherol (50 mg/Kg, Intraperitoneal (IP injection)) can attenuate temporary and permanent noise induced ABR threshold shifts and provide partially protection against ABR threshold shift resulting from exposure to noise plus CO in rabbits. Protective effect of *α*-tocopherol against combined exposure-induced hearing loss did not exceed that of noise alone.
